# Optimal solution of the fractional order breast cancer competition model

**DOI:** 10.1038/s41598-021-94875-1

**Published:** 2021-08-02

**Authors:** H. Hassani, J. A. Tenreiro Machado, Z. Avazzadeh, E. Safari, S. Mehrabi

**Affiliations:** 1 Department of Mathematics, Anand International College of Engineering, Jaipur, 302012 India; 2 Polytechnic of Porto, Dept. of Electrical Engineering, Institute of Engineering, R. Dr. António Bernardino de Almeida, Porto, 431 4249-015 Portugal; 3 Department of Applied Mathematics, Xi’an Jiaotong-Liverpool University, Suzhou, 215123 Jiangsu China; 4 Department of Immunology, School of Medicine, Iran University of Medical Sciences, Tehran, Iran; 5 Department of Internal Medicine, Shiraz University of Medical Sciences, Shiraz, Iran

**Keywords:** Breast cancer, Mathematics and computing

## Abstract

In this article, a fractional order breast cancer competition model (F-BCCM) under the Caputo fractional derivative is analyzed. A new set of basis functions, namely the generalized shifted Legendre polynomials, is proposed to deal with the solutions of F-BCCM. The F-BCCM describes the dynamics involving a variety of cancer factors, such as the stem, tumor and healthy cells, as well as the effects of excess estrogen and the body’s natural immune response on the cell populations. After combining the operational matrices with the Lagrange multipliers technique we obtain an optimization method for solving the F-BCCM whose convergence is investigated. Several examples show that a few number of basis functions lead to the satisfactory results. In fact, numerical experiments not only confirm the accuracy but also the practicability and computational efficiency of the devised technique.

## Introduction

Cancer is one of the most ubiquitous genetic diseases^1,2^. It is known that the disease arises because of mutations in the cancer susceptibility genes^3^. In order to understand the mechanism of human breast cancer various methods have been advanced. Loeb et al.^4^ proposed the existance of a mutator phenotype that acts as a mechanism in tumor processes. Tomlinson and Bordmer^5–7^ investigated the mutator phenotype hypothesis and discovered that the selection for clonal expansion of intermediate cells.

Mathematical models describe the fundamental principles of the population genetics and evolutionary mechanisms that govern tumor initiation and progression in cancer biology^8,9^. It was discovered that the dynamics of tumorgenesis is determined by a number of factors including mutation, selection, and tissue types^10–13^. It is also known that an increase in the postmenopausal and estrogen receptor-positive cases may increase the rate of breast cancer among women^14^. Several attempts have been made for the description of breast cancer in a variaty of perspectives. Enderling et al.^15^ presented a model of the growth and invasion of a solid tumour in a domain of breast tissue and proposed a scheme for the surgery and radiation treatment of the tumour. Enderling et al.^16^ applied surgery, as well as adjuvant external beam and targeted intraoperative radiotherapies, and used a model to identify different sources of local recurrence to analyse their prevention. Simmons et al.^17^ presented a brief overview of breast cancer, focussing on its heterogeneity and the role of modelling and simulation in teasing apart the underlying biophysical processes. Nave et al.^18^ described the treatment of breast cancer by a model involving nonlinear ordinary differential equations with a hidden hierarchy. Interested readers can follow also some recent works in^19–25^.

Fractional calculus is one of the most intensively developing branches of mathematical analysis and deals with derivatives and integrals of arbitrary order^26^. Fractional differential equations have motivated considerable attention in several branches of science^27–32^. In applied sciences, memory properties have been widely found in many complex phenomena. The use of fractional derivatives, instead of integer ones, can potencially lead to better results since one has an extra degree of freedom. Indeed, due to their intrinsic nonlocal property, the fractional differential equations have been successfully used to describe phenomena or processes with memory and hereditary properties in physics, chemistry, biology, and economy. Readers can refer to^33,34^. Fractional calculus is an excellent tool for modeling materials and processes with memory and hereditary properties and, in particular, electrochemical problems^35^. Fractional differentiation and integration operators are also used for generalizing the diffusion and wave equations^36,37^ or, more recently, of the temperature field problem in oil strata^38^. In what concerns cancer we can mention Farayola et al.^39^, that simulated a radiotherapy cancer treatment process with radiobiological factors, and Valentim et al.^40^, that propose a multistep exponential model with a fractional variable-order representing the evolution history of a tumor.

The Legendre polynomials are useful mathematical tools in fractional calculus. Cao et al.^41^ proposed a numerical algorithm based on the shifted Legendre polynomials to solve the fractional governing equations of polymethyl methacrylate in the time-domain. Zhijun et al.^42^ derived the operational matrix of fractional integration for Legendre polynomials to solve initial value problems of nonlinear fractional differential equations. Wang and Chen^43^ used the shifted Legendre polynomials for the dynamic analysis of viscoelastic pipes conveying fluid with variable order fractional model. Xiao et al.^44^ presented a finite-time empirical Gramians, constructed from impulse responses by solving block tridiagonal linear systems, to generate an approximate balanced model. Sun et al.^45^ designed an algorithm based on improved Legendre orthonormal basis for solving second-order boundary value problems. Hesameddini and Shahbazi^46^ approximated the unknown functions based on the two-dimensional shifted Legendre polynomials operational matrix method for the numerical solution of two-dimensional fractional integral equations. Guorong et al.^47^ proposed the Legendre orthogonal polynomials to calculate the acoustic reflection and transmission coefficients at liquid/solid interfaces. Singh et al.^48^ obtained numerical algorithms by using the Legendre, Galerkin and Legendre wavelet collocation methods for solving one phase moving boundary problem with conduction and convection effects. Rakhshan and Effati^49^ developed the generalized Legendre polynomials and derived a general procedure for solving nonlinear autonomous fractional differential equations with time varying delay. Heydari et al.^50^ presented a numerical method based on the discrete Legendre polynomials and the collocation scheme for solving nonlinear space-time fractional KdV-Burgers-Kuramoto equation. Kuznetsov^51^ used the Legendre polynomials and trigonometric functions for the numerical solution of the Ito stochastic differential equations when approximating multiple Ito and Stratonovich stochastic integrals based on generalized multiple Fourier series. Dehghan^52^ considered the shifted Legendre polynomials for class of variable order fractional functional boundary value problems.

In this work, an optimization method based on the generalized shifted Legendre polynomials (GSLP), operational matrix of derivatives and Lagrange multipliers is proposed for solving a fractional order breast cancer competition model (F-BCCM). The algorithm transforms the problem into a system of nonlinear algebraic equations with unknown coefficients, parameters and Lagrange multipliers. The optimal solution of the problem is obtained by solving an algebraic system of nonlinear equations. The results show that we can achieve the approximate solutions by employing only a few number of the basis functions. Moreover, the proposed approach can be adopted for solving other classes of fractional order problems.

The rest of the paper is structured as follows. Section 2 formulates the F-BCCM and the mathematical concept of fractional order Caputo derivative (F-CD). Section 3 discusses the shifted Legendre polynomials (SLP), as well as the GSLP and its operational matrix of derivatives and function approximation. Section 4 addresses the convergence analysis of the proposed method. Section 5 describes the method for finding the solution of the proposed problem. Section 6 discusses several illustrative examples. Section 7 analyses the epidemiologic and clinical relevance of the problem. Finally, Section 8 gives the main conclusions.

## Fractional order breast cancer competition model

The mathematical theory and concepts adressing the evolution of diseases and epidemics have been advanced during the last decades. Usually, these formulations consider that all people in a community starts as healthy and that later some of them may be diagnosed with breast (cancer). Abernathy et al.^19^ described the dynamic behavior of giving up BCCM. The model was discussed analytically and considering cancer stem cells (*C*), tumor cells (*T*), healthy cells (*H*), immune cells (*I*), and excess strogen (*E*). The proposed model is given as

2.1 $$\begin{aligned} \left\{ \begin{array}{lll} \frac{dC(t)}{dt}=k_{1}C(t)\left( 1-\frac{C(t)}{M_{1}}\right) -\gamma _{1}I(t)C(t)+\frac{p_{1}C(t)E(t)}{a_{1}+C(t)},\\ \frac{dT(t)}{dt}=k_{2}C(t)\left( \frac{C(t)}{M_{1}}\right) \left( 1-\frac{T(t)}{M_{2}}\right) -n_{1}T(t)-\gamma _{2}I(t)C(t)+\frac{p_{2}T(t)E(t)}{a_{2}+T(t)},\\ \frac{dH(t)}{dt}=qH(t)\left( 1-\frac{H(t)}{M_{3}}\right) -\delta H(t)T(t)-\frac{p_{3}H(t)E(t)}{a_{3}+H(t)},\\ \frac{dI(t)}{dt}=s+\frac{\rho I(t)T(t)}{\omega +T(t)}-\gamma _{3}I(t)T(t)-n_{2}I(t)-\frac{uI(t)E(t)}{v+E(t)},\\ \frac{dE(t)}{dt}=\tau -\left( \mu +\frac{d_{1}C(t)}{a_{1}+C(t)}+\frac{d_{2}T(t)}{a_{2}+T(t)}+\frac{d_{3}H(t)}{a_{3}+H(t)}\right) E(t),\\ C_{0}(t)=C(0),\,\,\,\,\,T_{0}(t)=T(0),\,\,\,\,\,H_{0}(t)=H(0),\,\,\,\,\,I_{0}(t)=I(0),\,\,\,\,\,E_{0}(t)=E(0). \end{array} \right. \end{aligned}$$

In Table 1, we list the description and baseline values of the parameters in the system (2.1).

**Table 1:** The parameters of the BCCM model.

Parameters	Description	Value
$k_{1}$	Normal rate of division for *C* cells	0.75 $\hbox {day}^{-1}$
$k_{2}$	Normal rate of division for *T* cells	0.514 $\hbox {day}^{-1}$
*q*	Normal rate of division for *H* cells	0.70 $\hbox {day}^{-1}$
$M_{1}$	Carrying capacity of *C* cells	$2.27\times 10^{6}$ cells
$M_{2}$	Carrying capacity of *T* cells	$2.27\times 10^{7}$ cells
$M_{3}$	Carrying capacity of *H* cells	$2.5\times 10^{7}$ cells
$\gamma _{1}$	Cancer stem cell death rate due to immune cell response	$3\times 10^{-7}$ $\hbox {cell}^{-1}$ $\hbox {day}^{-1}$
$\gamma _{2}$	Tumor cell death rate due to immune cell response	$3\times 10^{-6}$ $\hbox {cell}^{-1}$ $\hbox {day}^{-1}$
$\gamma _{3}$	Immune cell death rate due to tumor cell response	$1\times 10^{-7}$ $\hbox {cell}^{-1}$ $\hbox {day}^{-1}$
$p_{1}$	Rate at which estrogen helps to proliferate cancer stem cells	600 cell $\hbox {day}^{-1}$ (pg/mL)$^{-1}$
$p_{2}$	Rate at which estrogen helps to proliferate tumor cells	0 cell $\hbox {day}^{-1}$ (pg/mL)$^{-1}$
$p_{3}$	Rate at which healthy cells are lost to DNA mutation by estrogen presence	100 cell $\hbox {day}^{-1}$ (pg/mL)$^{-1}$
$a_{1}$	Number of *C* cells at which the rate of absorption is at half its maximum	$\frac{1}{2}M_{1}$ cells
$a_{2}$	Number of *T* cells at which the rate of absorption is at half its maximum	$\frac{1}{2}M_{2}$ cells
$a_{3}$	Number of *H* cells at which the rate of absorption is at half its maximum	$\frac{1}{2}M_{3}$ cells
$n_{1}$	Normal death rate of tumor cells	0.01 $\hbox {day}^{-1}$
$n_{2}$	Normal death rate of immune cells	0.29 $\hbox {day}^{-1}$
$\delta$	Healthy cell death rate due to competition with tumor cells	$6\times 10^{-8}$ $\hbox {day}^{-1}$ $\hbox {cell}^{-1}$
*s*	Source rate of immune cells	$1.3\times 10^{4}$ cell $\hbox {day}^{-1}$
$\rho$	Immune cell response rate	0.20 $\hbox {day}^{-1}$
$\omega$	Immune cell threshold	$3\times 10^{5}$ cells
*u*	Rate of immune suppression by estrogen	0.20 $\hbox {day}^{-1}$
*v*	Estrogen threshold	400 pg $\hbox {mL}^{-1}$
$\tau$	Continuous infusion of estrogen	2000 pg $\hbox {mL}^{-1}$ $\hbox {day}^{-1}$
$\mu$	Washout rate of estrogen by the body	0.97 $\hbox {day}^{-1}$
$d_{1}$	Absorption rate of estrogen by cancer stem cells	0.01 $\hbox {day}^{-1}$
$d_{2}$	Absorption rate of estrogen by tumor cells	0.01 $\hbox {day}^{-1}$
$d_{3}$	Absorption rate of estrogen by healthy cells	0.01 $\hbox {day}^{-1}$

This paper formulates an alternative representation of the BCCM considering the F-CD. The F-CD of order $0<\eta \le 1$, with respect to *t* is given by^53,54^:

2.2 $$\begin{aligned} ^{C}_{0}{D_{t}^{\eta }}f(t)=\left\{ \begin{array}{lll} \frac{1}{\Gamma \left( 1-\eta \right) }\int _{0}^{t}\left( t-\xi \right) ^{-\eta } f^{\prime }(\xi )d\xi ,\quad &{}&{}0<\eta < 1,\\ \frac{df(t)}{dt},\quad &{}&{}\eta =1, \end{array} \right. \end{aligned}$$

where $\Gamma (\cdot )$ denotes the Gamma function $\Gamma (z)=\int _{0}^{\infty }t^{z-1}e^{-t}dt, z>0$. In expression (2.2), the convolution integral represents the memory effect embedded in the fractional derivative. Indeed, the fractional derivative uses the previous values of *f*(*t*), and captures the long memory effect of the dynamics. From (2.2), for any $r\in {\mathbb {N}}$, we can write

2.3 $$\begin{aligned} ^{C}_{0}{D_{t}^{\eta }}t^{r}= \displaystyle \left\{ \begin{array}{ll} \displaystyle \frac{\Gamma (r+1)}{\Gamma (r-\eta +1)}\,t^{r-\eta }, &{} r=1,2,\ldots , \\ 0, &{} r=0. \end{array} \right. \end{aligned}$$

Rewriting the model (2.1) in terms of the F-CD, we obtain

2.4 $$\begin{aligned} \left\{ \begin{array}{lll} ^{C}_{0}{D_{t}^{\eta _{1}}}C(t)=k_{1}C(t)\left( 1-\frac{C(t)}{M_{1}}\right) -\gamma _{1}I(t)C(t)+\frac{p_{1}C(t)E(t)}{a_{1}+C(t)},\\ ^{C}_{0}{D_{t}^{\eta _{2}}}T(t)=k_{2}C(t)\left( \frac{C(t)}{M_{1}}\right) \left( 1-\frac{T(t)}{M_{2}}\right) -n_{1}T(t)-\gamma _{2}I(t)C(t)+\frac{p_{2}T(t)E(t)}{a_{2}+T(t)},\\ ^{C}_{0}{D_{t}^{\eta _{3}}}H(t)=qH(t)\left( 1-\frac{H(t)}{M_{3}}\right) -\delta H(t)T(t)-\frac{p_{3}H(t)E(t)}{a_{3}+H(t)},\\ ^{C}_{0}{D_{t}^{\eta _{4}}}I(t)=s+\frac{\rho I(t)T(t)}{\omega +T(t)}-\gamma _{3}I(t)T(t)-n_{2}I(t)-\frac{uI(t)E(t)}{v+E(t)},\\ ^{C}_{0}{D_{t}^{\eta _{5}}}E(t)=\tau -\left( \mu +\frac{d_{1}C(t)}{a_{1}+C(t)}+\frac{d_{2}T(t)}{a_{2}+T(t)}+\frac{d_{3}H(t)}{a_{3}+H(t)}\right) E(t),\\ C_{0}(t)=C(0),\,\,\,\,\,T_{0}(t)=T(0),\,\,\,\,\,H_{0}(t)=H(0),\,\,\,\,\,I_{0}(t)=I(0),\,\,\,\,\,E_{0}(t)=E(0), \end{array} \right. \end{aligned}$$

where $^{C}_{0}{D_{t}^{\eta _{i}}}$, $i = 1,2,3,4,5$, represents the F-CD of order $0<\eta _{i}\le 1$.

We must note that the dimension of the left-side equations of model (2.4) is (time)$^{-\eta _{i}}$, $i = 1,2,3,4,5$. Nonetheless, a close inspection of the right-hand sides shows that the quantities $k_{1}$, $k_{2}$, *q*, $\gamma _{1}$, $\gamma _{2}$, $\gamma _{3}$, $p_{1}$, $p_{2}$, $p_{3}$, $n_{1}$, $n_{2}$, $\delta$, *s*, $\rho$, *u*, $\tau$, $\mu$, $d_{1}$, $d_{2}$ and $d_{3}$ have the dimension (time)$^{-1}$ and, therefore, we need to modify the right-hand sides to match the dimensions. The most straightforward way of doing this gives the following model

2.5 $$\begin{aligned} \left\{ \begin{array}{lll} ^{C}_{0}{D_{t}^{\eta _{1}}}C(t)=k_{1}^{\eta _{1}}C(t)\left( 1-\frac{C(t)}{M_{1}}\right) -\gamma _{1}^{\eta _{1}}I(t)C(t)+\frac{p_{1}^{\eta _{1}}C(t)E(t)}{a_{1}+C(t)},\\ ^{C}_{0}{D_{t}^{\eta _{2}}}T(t)=k_{2}^{\eta _{2}}C(t)\left( \frac{C(t)}{M_{1}}\right) \left( 1-\frac{T(t)}{M_{2}}\right) -n_{1}^{\eta _{2}}T(t)-\gamma _{2}^{\eta _{2}}I(t)C(t)+\frac{p_{2}^{\eta _{2}}T(t)E(t)}{a_{2}+T(t)},\\ ^{C}_{0}{D_{t}^{\eta _{3}}}H(t)=q^{\eta _{3}}H(t)\left( 1-\frac{H(t)}{M_{3}}\right) -\delta ^{\eta _{3}} H(t)T(t)-\frac{p_{3}^{\eta _{3}}H(t)E(t)}{a_{3}+H(t)},\\ ^{C}_{0}{D_{t}^{\eta _{4}}}I(t)=s^{\eta _{4}}+\frac{\rho ^{\eta _{4}} I(t)T(t)}{\omega +T(t)}-\gamma _{3}^{\eta _{4}}I(t)T(t)-n_{2}^{\eta _{4}}I(t)-\frac{u^{\eta _{4}}I(t)E(t)}{v+E(t)},\\ ^{C}_{0}{D_{t}^{\eta _{5}}}E(t)=\tau ^{\eta _{5}}-\left( \mu ^{\eta _{5}}+\frac{d_{1}^{\eta _{5}}C(t)}{a_{1}+C(t)}+\frac{d_{2}^{\eta _{5}}T(t)}{a_{2}+T(t)}+\frac{d_{3}^{\eta _{5}}H(t)}{a_{3}+H(t)}\right) E(t),\\ C_{0}(t)=C(0),\,\,\,\,\,T_{0}(t)=T(0),\,\,\,\,\,H_{0}(t)=H(0),\,\,\,\,\,I_{0}(t)=I(0),\,\,\,\,\,E_{0}(t)=E(0). \end{array} \right. \end{aligned}$$

This is the system that we will actually use for modeling our problem. Note that in the limit case $\eta _{i}\longrightarrow 1$, $i = 1,2,3,4,5$, the system (2.5) reduces to classical one (2.1)^55,56^.

## Basis functions

Hereafter, we introduce two classes of the basis functions, namelly the SLP and GSLP, which will be used in approximating solutions of the F-BCCM (2.4).

### Approximation by the shifted Legendre polynomials

The Legendre polynomials, defined on the interval $[-1,1]$, can be determined with the recurrence formula

3.1 $$\begin{aligned} P_{j+1}(t)=\frac{2j+1}{j+1}t P_{j}(t)-\frac{j}{j+1}P_{j-1}(t),j=1,2,\ldots , \end{aligned}$$

where $P_{0}(t)=1$ and $P_{1}(t)=t$. To use Legendre polynomials in the interval [0, 1] we have to define the SLP by means of the change of variable $t\rightarrow 2t-1$. The SLP, $P_{j}(2t-1)$, can be denoted by $L_{j}(t)$. Therefore, $L_{j}(t)$ follows the relationship:

3.2 $$\begin{aligned} L_{j+1}(t)=\frac{(2j+1)(2t-1)}{j+1}L_{j}(t)-\frac{j}{j+1}L_{j-1}(t),j=1,2,\ldots , \end{aligned}$$

where $L_{0}(t)=1$ and $L_{1}(t)=2t-1$. The analytical form of the SLP of degree *j*, $L_{j}(t)$, is given by:

3.3 $$\begin{aligned} L_{j}(t)=\sum _{k=0}^{j}(-1)^{j+k}\frac{(j+k)!}{(j-k)!}\frac{t^{k}}{(k!)^{2}}. \end{aligned}$$

Note that $L_{j}(0)=(-1)^{j}$ and $L_{j}(1)=1$.

A given function *g*(*t*) can be expressed using the SLP as follows

3.4 $$\begin{aligned} g(t)={\mathscr {R}}^{T}{\mathscr {Q}}_{n}(t)={\mathscr {R}}^{T}{\mathscr {P}}\,\Phi _{n}(t), \end{aligned}$$

where ${\mathscr {Q}}_{n}(t)$ is an $(n+1)$-order column vector including the basis functions. The following selections for ${\mathscr {R}}^{T}$ and ${\mathscr {Q}}_{n}(t)$ are considered as

3.5 $$\begin{aligned} {\mathscr {R}}^{T}=[r_{0}\,\,r_{1}\,\,\ldots \,\,r_{n}],\,\,\,\,\,\,\ {\mathscr {P}}= \begin{pmatrix} p_{0,0} &{} p_{0,1}&{}\cdots &{}p_{0,n} \\ p_{1,0} &{} p_{1,1}&{}\cdots &{}q_{1,n} \\ \vdots &{} \vdots &{} \cdots &{}\vdots \\ p_{n,0} &{} p_{n,1}&{}\cdots &{} p_{n,n} \\ \end{pmatrix},\,\,\,\,\,\,\, \Phi _{n}(t)=[1\,\,\,t\,\,\,t^{2}\,\,\,\ldots \,t^{n}]^{T}, \end{aligned}$$

and

3.6 $$\begin{aligned} p_{ij}= \displaystyle \left\{ \begin{array}{ll} \displaystyle (-1)^{i+j}\frac{(i+j)!}{(i-j)!(j!)^{2}}, &{} i\ge j, \\ 0, &{} i<j, \end{array} \right. i,j=0,1,\ldots ,n. \end{aligned}$$

### Approximation by the generalized shifted Legendre polynomials

Let us define a set of basis functions based on the GSLP to obtain an efficient solution of (2.4). For $m\in {\mathbb {N}}$, the GSLP, $\mathcal {L}_{m}(t)$, are constructed through a change of variable. Therefore, $t^{i}$ is transformed into $t^{i+\alpha _{i}}$, $i+\alpha _{i} > 0$, in the SLP and are defined by

3.7 $$\begin{aligned} \mathcal {L}_{m}(t)=\sum _{i=0}^{m}(-1)^{n+i}\frac{(n+i)!}{(n-i)!}\frac{t^{i+\alpha _{i}}}{(i!)^{2}}, \end{aligned}$$

where $\alpha _{i}$ denotes the control parameters. If $\alpha _{i}=0$, then the GSLP coincide with the classical SLP.

By using the GSLP, the functions *C*(*t*), *T*(*t*), *H*(*t*), *I*(*t*) and *E*(*t*) can be expressed in the following form:

3.8 $$\begin{aligned} C(t)= & {} {\mathscr {A}}^{T}~\mathcal {Q}^{1}~\Psi ^{1}(t),\quad T(t)={\mathscr {B}}^{T}~\mathcal {Q}^{2}~\Psi ^{2}(t),\quad H(t)={\mathscr {C}}^{T}~\mathcal {Q}^{3}~\Psi ^{3}(t),\nonumber \\ I(t)= & {} {\mathscr {D}}^{T}~\mathcal {Q}^{4}~\Psi ^{4}(t),\quad E(t)={\mathscr {E}}^{T}~\mathcal {Q}^{5}~\Psi ^{5}(t), \end{aligned}$$

where

3.9 $$\begin{aligned} \mathcal {Q}^{1}= & {} \begin{pmatrix} 1 &{} 0&{}0&{}\cdots &{} 0 \\ q_{1,0}^{1} &{} q_{1,1}^{1}&{}q_{1,2}^{1}&{}\cdots &{}q_{1,m_{1}}^{1} \\ q_{2,0}^{1} &{} q_{2,1}^{1}&{}q_{2,2}^{1}&{}\cdots &{}q_{2,m_{1}}^{1} \\ \vdots &{} \vdots &{}\vdots &{} \cdots &{}\vdots \\ q_{m_{1},0}^{1} &{} q_{m_{1},1}^{1}&{}q_{m_{1},2}^{1}&{}\cdots &{} q_{m_{1},m_{1}}^{1} \\ \end{pmatrix},\quad \mathcal {Q}^{2}= \begin{pmatrix} 1 &{} 0&{}0&{}\cdots &{} 0 \\ q_{1,0}^{2} &{} q_{1,1}^{2}&{}q_{1,2}^{2}&{}\cdots &{}q_{1,m_{2}}^{2} \\ q_{2,0}^{2} &{} q_{2,1}^{2}&{}q_{2,2}^{2}&{}\cdots &{}q_{2,m_{2}}^{2} \\ \vdots &{} \vdots &{}\vdots &{} \cdots &{}\vdots \\ q_{m_{2},0}^{2} &{} q_{m_{2},1}^{2}&{}q_{m_{2},2}^{2}&{}\cdots &{} q_{m_{2},m_{2}}^{2} \\ \end{pmatrix}, \end{aligned}$$

3.10 $$\begin{aligned} \mathcal {Q}^{3}= & {} \begin{pmatrix} 1 &{} 0&{}0&{}\cdots &{} 0 \\ q_{1,0}^{3} &{} q_{1,1}^{3}&{}q_{1,2}^{3}&{}\cdots &{}q_{1,m_{3}}^{3} \\ q_{2,0}^{3} &{} q_{2,1}^{3}&{}q_{2,2}^{3}&{}\cdots &{}q_{2,m_{3}}^{3} \\ \vdots &{} \vdots &{}\vdots &{} \cdots &{}\vdots \\ q_{m_{3},0}^{3} &{} q_{m_{3},1}^{3}&{}q_{m_{3},2}^{3}&{}\cdots &{} q_{m_{3},m_{3}}^{3} \\ \end{pmatrix},\quad \mathcal {Q}^{4}= \begin{pmatrix} 1 &{} 0&{}0&{}\cdots &{} 0 \\ q_{1,0}^{4} &{} q_{1,1}^{4}&{}q_{1,2}^{4}&{}\cdots &{}q_{1,m_{4}}^{4} \\ q_{2,0}^{4} &{} q_{2,1}^{4}&{}q_{2,2}^{4}&{}\cdots &{}q_{2,m_{4}}^{4} \\ \vdots &{} \vdots &{}\vdots &{} \cdots &{}\vdots \\ q_{m_{4},0}^{4} &{} q_{m_{4},1}^{4}&{}q_{m_{4},2}^{4}&{}\cdots &{} q_{m_{4},m_{4}}^{4} \\ \end{pmatrix}, \end{aligned}$$

3.11 $$\begin{aligned} \mathcal {Q}^{5}= & {} \begin{pmatrix} 1 &{} 0&{}0&{}\cdots &{} 0 \\ q_{1,0}^{5} &{} q_{1,1}^{5}&{}q_{1,2}^{5}&{}\cdots &{}q_{1,m_{5}}^{5} \\ q_{2,0}^{5} &{} q_{2,1}^{5}&{}q_{2,2}^{5}&{}\cdots &{}q_{2,m_{5}}^{5} \\ \vdots &{} \vdots &{}\vdots &{} \cdots &{}\vdots \\ q_{m_{5},0}^{5} &{} q_{m_{5},1}^{5}&{}q_{m_{5},2}^{5}&{}\cdots &{} q_{m_{5},m_{5}}^{5} \\ \end{pmatrix}, \end{aligned}$$

3.12 $$\begin{aligned} {\mathscr {A}}^{T}= & {} [a_{0}~~a_{1}~~\ldots ~~a_{m_{1}}],\quad {\mathscr {B}}^{T}=[b_{0}~~b_{1}~~\ldots ~~b_{m_{2}}],\quad {\mathscr {C}}^{T}=[c_{0}~~c_{1}~~\ldots ~~c_{m_{3}}],\nonumber \\ {\mathscr {D}}^{T}= & {} [d_{0}~~d_{1}~~\ldots ~~d_{m_{4}}],\quad {\mathscr {E}}^{T}=[e_{0}~~e_{1}~~\ldots ~~e_{m_{5}}],\nonumber \\ \Psi ^{1}(t)\triangleq & {} [\psi ^{1}_{0}(t)\,\,\,\psi ^{1}_{1}(t)\,\,\,\ldots \,\psi ^{1}_{m_{1}}(t)]^{T},\quad \Psi ^{2}(t)\triangleq [\psi ^{2}_{0}(t)\,\,\,\psi ^{2}_{2}(t)\,\,\,\ldots \,\psi ^{2}_{m_{2}}(t)]^{T},\nonumber \\ \Psi ^{3}(t)\triangleq & {} [\psi ^{3}_{0}(t)\,\,\,\psi ^{3}_{1}(t)\,\,\,\ldots \,\psi ^{3}_{m_{3}}(t)]^{T},\quad \Psi ^{4}(t)\triangleq [\psi ^{4}_{0}(t)\,\,\,\psi ^{4}_{2}(t)\,\,\,\ldots \,\psi ^{4}_{m_{4}}(t)]^{T},\nonumber \\ \Psi ^{5}(t)\triangleq & {} [\psi ^{5}_{0}(t)\,\,\,\psi ^{5}_{1}(t)\,\,\,\ldots \,\psi ^{5}_{m_{5}}(t)]^{T} \end{aligned}$$

and

3.13 $$\begin{aligned} q_{kj}^{i}= & {} \displaystyle \left\{ \begin{array}{ll} \displaystyle (-1)^{k+j}\frac{(k+j)!}{(k-j)!(j!)^{2}}, &{} k\ge j, \\ 0, &{} k<j, \end{array} \right. \quad k=1,2,\ldots , m_{i},\quad j=0,1,\ldots , m_{i},\quad i=1,2,\ldots , 5, \end{aligned}$$

3.14 $$\begin{aligned} \psi ^{i}_{j}(t)= & {} \left\{ \begin{array}{lll} 1,&{}&{}j=0,\\ t^{j+\alpha ^{i}_{j}},&{}&{}j=1,2,\ldots ,m_{i}, \end{array} \right. \quad i=1,2,\ldots , 5, \end{aligned}$$

with $\alpha ^{i}_{j}$ standing for the control parameters.

The $\eta _{i}$-th F-CD of $\Psi ^{i}(t), ~ i=1,2,\ldots ,5,$ is given by

3.15 $$\begin{aligned} ^{C}_{0}{D_{t}^{\eta _{i}}}\Psi ^{i}(t)=\mathcal {D}^{\left( \eta _{i}\right) }_{t}~\Psi ^{i}(t), \end{aligned}$$

where $\mathcal {D}^{\left( \eta _{i}\right) }_{t}$ (the operational matrix of F-CD of order $\eta _{i}$) is as follows

3.16 $$\begin{aligned} \mathcal {D}^{\left( \eta _{i}\right) }_{t}=t^{-\eta _{i}} \begin{pmatrix} 0&{}0&{}0&{}\cdots &{}0 \\ 0&{}\frac{\Gamma \left( 2+\alpha ^{i}_{1}\right) }{\Gamma \left( 2-\eta _{i}+\alpha ^{i}_{1}\right) }&{}0&{}\cdots &{}0 \\ 0&{}0&{}\frac{\Gamma \left( 3+\alpha ^{i}_{2}\right) }{\Gamma \left( 3-\eta _{i}+\alpha ^{i}_{2}\right) }&{}\cdots &{}0 \\ \vdots &{}\vdots &{}\vdots &{}\ddots &{}\vdots \\ 0&{}0&{}0&{}\cdots &{}\frac{\Gamma \left( m_{i}+1+\alpha ^{i}_{m_{i}}\right) }{\Gamma \left( m_{i}+1-\eta _{i}+\alpha ^{i}_{m_{i}}\right) } \\ \end{pmatrix}. \end{aligned}$$

### Function approximation

Let us consider $\mathcal {X}=L^{2}[0,1]$. We introduce

$$\begin{aligned} \mathcal {Y}_{m_{1}}=span \{1\,\,\,\mathcal {L}_{1}(t)\,\,\,\mathcal {L}_{2}(t)\,\,\,\ldots \,\,\,\,\mathcal {L}_{m_{1}}(t)\}. \end{aligned}$$

Let $u^{*}(t)\in \mathcal {Y}_{m_{1}}$ be the best approximation of *u*(*t*). Therefore, we have

$$\begin{aligned} \forall ~v\in \mathcal {Y}_{m_{1}},~~~\parallel u-u^{*}\parallel _{2}\le \parallel u-v\parallel _{2}. \end{aligned}$$

Since $u^{*}\in \mathcal {Y}_{m_{1}}$, there exist the unique coefficients ${\mathscr {A}}^{T}=[a_{0}~~a_{1}~~\ldots ~~a_{m_{1}}]$, such that

$$\begin{aligned} A(t)\simeq u^{*}(t)={\mathscr {A}}^{T}~\mathcal {Q}^{1}~\Psi ^{1}(t), \end{aligned}$$

where $\mathcal {Q}^{1}$ and $\Psi ^{1}(t)$ are defined in Eqs. (3.9) and (3.12).

## Convergence analysis

The classical Weierstrass theorem (see^57^, Theorem 7.26) states that polynomials are dense in *C*(*I*), the space of all continuous complex functions on the closed interval $I=[0,1]$ with the supremum norm. In other words, the set of all finite linear combinations of the functions

4.1 $$\begin{aligned} 1, t, t^2, t^3,\ldots , \end{aligned}$$

is dense in *C*(*I*). This can be expressed by saying that the functions (4.1) span *C*(*I*). The following question arises naturally, namely, if $0<\lambda _1<\lambda _2<\cdots$, under what conditions, then is it true that the functions

4.2 $$\begin{aligned} 1,t^{\lambda _1},t^{\lambda _2},\ldots , \end{aligned}$$

span *C*(*I*)? The neat answer is that the functions (4.2) span *C*(*I*) if and only if $\sum _{n=1}^{\infty }\frac{1}{\lambda _n}=\infty$.

### Theorem 1

(The Müntz-Szasz Theorem):^58^ *Suppose that* $0<\lambda _1<\lambda _2<\cdots$ *and let* *X* *be the closure in* *C*(*I*) *of the set of all finite linear combinations of the functions*

4.3 $$\begin{aligned} 1,t^{\lambda _1},t^{\lambda _2},\ldots . \end{aligned}$$

- *If* $$\sum _{n=1}^{\infty }\frac{1}{\lambda _n}=\infty$$, *then* $$X=C(I)$$.
- *If* $\sum _{n=1}^{\infty }\frac{1}{\lambda _n}<\infty$ *and if* $\lambda \notin \{\lambda _n\}_{n\in \mathbb {N}}$, $\lambda \ne 0$, *then* *X* *does not contain the function* $t^{\lambda _n}$.

### Remark 1

Let ${{\mathcal {C}}}_{\lambda }(I)\subset C(I)$ be the set of all finite linear combinations of the functions defined in (4.2), where $0<\lambda _1<\lambda _2<\cdots$ and let ${{\mathcal {C}}}_{\beta }(I)$ be the set of the functions

4.4 $$\begin{aligned} 1, t^{1+\beta _1}, t^{2+\beta _2}, t^{3+\beta _3},\ldots ,t^{i+\beta _i},\ldots , \end{aligned}$$

where $\{\beta _i\}_{i\in \mathbb {N}}$ is a sequence of real numbers, with $i+\beta _i\ge 0$ for all $i\in \mathbb {N}$. It is obvious that ${{\mathcal {C}}}_{\lambda }(I)\subset {{\mathcal {C}}}_{\beta }(I)$ and, hence, $\overline{{{\mathcal {C}}}_{\lambda }(I)}\subset \overline{{{\mathcal {C}}}_{\beta }(I)}$, where $\overline{{{\mathcal {C}}}_{\lambda }(I)}$ and $\overline{{{\mathcal {C}}}_{\beta }(I)}$ are the closures of the sets ${{\mathcal {C}}}_{\lambda }(I)$ and ${{\mathcal {C}}}_{\beta }(I)$ in *C*(*I*), respectively. In view of Theorem 1, it is apparent that the set ${{\mathcal {C}}}_{\beta }(I)$ is dense in *C*(*I*), i.e., $\overline{{{\mathcal {C}}}_{\lambda }(I)}=C(I)$.

Similarly, if ${{\mathcal {C}}}_{\gamma }(I)$ is the set of set of all finite linear combinations of the functions

4.5 $$\begin{aligned}&1, -\frac{(n+1)!}{(n-1)(1!)^2}t^{1+\gamma _1}, \frac{(n+2)!}{(n-2)(2!)^2}t^{2+\gamma _2},\nonumber \\&\quad -\frac{(n+3)!}{(n-3)(3!)^2}t^{3+\gamma _3},\ldots ,(-1)^{n+i}\frac{(n+i)!}{(n-i)(i!)^2}t^{i+\gamma _i},\ldots , \end{aligned}$$

where $\{\gamma _i\}_{i\in \mathbb {N}}$ is a sequence of real numbers, with $i+\gamma _i\ge 0$ for all $i\in \mathbb {N}$, then, in view of Theorem 1, the set ${{\mathcal {C}}}_{\gamma }(I)$ is dense in *C*(*I*), i.e., $\overline{{{\mathcal {C}}}_{\gamma }(I)}=C(I)$.

The following two theorems are immediate consequences of Theorem 1 and, therefore, we omit the details.

### Theorem 2

*Suppose that* $0<1+\beta _1<2+\beta _2<\cdots$ *and let* ${{\mathcal {C}}}_{\beta }(I)$ *be the set of all finite linear combinations of the functions defined in* (4.4).

- *If* $\sum _{n=1}^{\infty }\frac{1}{n+\beta _n}=\infty$, *then* $Y:=\overline{{{\mathcal {C}}}_{\beta }(I)}=C(I)$, *where* $\overline{{{\mathcal {C}}}_{\beta }(I)}$ *is the closure of the set* ${{\mathcal {C}}}_{\beta }(I)$ in *C*(*I*).
- *If* $\sum _{n=1}^{\infty }\frac{1}{n+\beta _n}<\infty$, *and* *if* $\beta \notin \{\beta _n\}_{n\in \mathbb {N}}$, $\beta \ne 0$, *then* *Y* *does not contain the function* $t^{\beta _n}$.

### Theorem 3

*Suppose that* $0<1+\gamma _1<2+\gamma _2<\cdots$ *and let* ${{\mathcal {C}}}_{\gamma }(I)$ *be the set of all finite linear combinations of the functions defined in* (4.5).

- *If* $\sum _{n=1}^{\infty }\frac{1}{n+\gamma _n}=\infty$, *then* $Z:=\overline{{{\mathcal {C}}}_{\gamma }(I)}=C(I)$, *where* $\overline{{{\mathcal {C}}}_{\gamma }(I)}$ *is the closure of the set* ${{\mathcal {C}}}_{\gamma }(I)$ in *C*(*I*).
- *If* $\sum _{n=1}^{\infty }\frac{1}{n+\gamma _n}<\infty$, *and if* $\gamma \notin \{\gamma _n\}_{n\in \mathbb {N}}$, $\gamma \ne 0$, *then* *Z* *does not contain the function* $t^{\gamma _n}$.

*We now investigate the convergence of the proposed method. We first discuss the convergence analysis of the GP expansion by means of the following theorem.*

### Theorem 4

*Let* $f:[0,1]\rightarrow {\mathbb {R}}$ *be a continuous function. Then, for every* $\epsilon >0$ *there exists a generalized polynomials*^59^, ${\mathscr {P}}_{m_1}(t)$, *such that*

$$\begin{aligned} \Vert f-{\mathscr {P}}_{m_1}\Vert =\sup \{|f(t)-{\mathscr {P}}_{m_1}(t)|:t\in [0,1]\}<\epsilon . \end{aligned}$$

### Proof

Let $\epsilon >0$ be a fixed real number. Chose a sequence $\{\beta _i\}_{i\in \mathbb {N}}$ of real numbers with $i+\beta _i\ge 0$, $0<i+\beta _i<i+1+\beta _{i+1}$ for all $i\in \mathbb {N}$ and $\sum _{n=1}^{\infty }\frac{1}{n+\beta _n}=\infty$. Applying Theorem 1, we get the desired result. This completes the proof.

For discussing the convergence analysis of the GSLP expansion we consider the following theorem. $\square$

### Theorem 5

*Let* $f:[0,1]\rightarrow {\mathbb {R}}$ *be a continuous function. Then, for every* $\epsilon >0$, *there exists a GSLP,* ${\mathscr {L}}_{m_1}(t)$, *such that*

$$\begin{aligned} \Vert f-{\mathscr {L}}_{m_1}\Vert =\sup \{|f(t)-{\mathscr {L}}_{m_1}(t)|:t\in [0,1]\}<\epsilon . \end{aligned}$$

### Proof

Let $\epsilon >0$ be a fixed real number. Chose a sequence $\{\gamma _i\}_{i\in \mathbb {N}}$ of real numbers with $i+\gamma _i\ge 0$, $0<i+\gamma _i<i+1+\gamma _{i+1}$ for all $i\in \mathbb {N}$ and $\sum _{n=1}^{\infty }\frac{1}{n+\gamma _n}=\infty$. Applying Theorem 1, we get the desired result. This completes the proof. $\square$

## The proposed strategy

In this section, we design a matrix approach by using the GSLP to solve the problem generated in Eq. (2.5). To carry out this method, we approximate *C*(*t*), *T*(*t*), *H*(*t*), *I*(*t*) and *E*(*t*) by the GSLP basis as follows

5.1 $$\begin{aligned} C(t)= & {} {\mathscr {A}}^{T}~\mathcal {Q}^{1}~\Psi ^{1}(t),\quad T(t)={\mathscr {B}}^{T}~\mathcal {Q}^{2}~\Psi ^{2}(t),\quad H(t)={\mathscr {C}}^{T}~\mathcal {Q}^{3}~\Psi ^{3}(t),\nonumber \\ I(t)= & {} {\mathscr {D}}^{T}~\mathcal {Q}^{4}~\Psi ^{4}(t),\quad E(t)={\mathscr {E}}^{T}~\mathcal {Q}^{5}~\Psi ^{5}(t), \end{aligned}$$

where ${\mathscr {A}}^{T}$, ${\mathscr {B}}^{T}$, ${\mathscr {C}}^{T}$, ${\mathscr {D}}^{T}$, ${\mathscr {E}}^{T}$ and $\Phi ^{i}=\left[ \alpha ^{i}_{1}\,\ \alpha ^{i}_{2}\,\ldots \,\alpha ^{i}_{m_{i}}\right]$, $i=1,2,3,4,5$, are undetermined vectors including the free coefficients and control parameters, and $\mathcal {Q}^{i}$ and $\Psi ^{i}(t)$, $i=1,2,3,4,5$, are defined in Eqs. (3.9–3.14). Regarding (3.16), one has

5.2 $$\begin{aligned} ^{C}_{0}{D_{t}^{\eta _{1}}}C(t)= & {} {\mathscr {A}}^{T}~\mathcal {Q}^{1}~\mathcal {D}^{\left( \eta _{1}\right) }_{t}~\Psi ^{1}(t),\nonumber \\ ^{C}_{0}{D_{t}^{\eta _{2}}}T(t)= & {} {\mathscr {B}}^{T}~\mathcal {Q}^{2}~\mathcal {D}^{\left( \eta _{2}\right) }_{t}~\Psi ^{2}(t),\nonumber \\ ^{C}_{0}{D_{t}^{\eta _{3}}}H(t)= & {} {\mathscr {C}}^{T}~\mathcal {Q}^{3}~\mathcal {D}^{\left( \eta _{3}\right) }_{t}~\Psi ^{3}(t),\nonumber \\ ^{C}_{0}{D_{t}^{\eta _{4}}}I(t)= & {} {\mathscr {D}}^{T}~\mathcal {Q}^{4}~\mathcal {D}^{\left( \eta _{4}\right) }_{t}~\Psi ^{4}(t),\nonumber \\ ^{C}_{0}{D_{t}^{\eta _{5}}}E(t)= & {} {\mathscr {E}}^{T}~\mathcal {Q}^{5}~\mathcal {D}^{\left( \eta _{5}\right) }_{t}~\Psi ^{5}(t). \end{aligned}$$

Moreover, we approximate the initial conditions given in Eq. (2.5) via the GSLP as follows

5.3 $$\begin{aligned} C(0)\simeq & {} {\mathscr {A}}^{T}~\mathcal {Q}^{1}~\Psi ^{1}(0),\quad T(0)\simeq {\mathscr {B}}^{T}~\mathcal {Q}^{2}~\Psi ^{2}(0),\quad H(0)\simeq {\mathscr {C}}^{T}~\mathcal {Q}^{3}~\Psi ^{3}(0),\nonumber \\ I(0)\simeq & {} {\mathscr {D}}^{T}~\mathcal {Q}^{4}~\Psi ^{4}(0),\quad E(0)\simeq {\mathscr {E}}^{T}~\mathcal {Q}^{5}~\Psi ^{5}(0). \end{aligned}$$

Now, we define the residual function by using Eq. (2.5) and Eqs. (5.1–5.2), so that

5.4 $$\begin{aligned} \left\{ \begin{array}{ll} {\mathscr {R}}_{1}(t)={\mathscr {A}}^{T}~\mathcal {Q}^{1}~\mathcal {D}^{\left( \eta _{1}\right) }_{t}~\Psi ^{1}(t)-k_{1}^{\eta _{1}}{\mathscr {A}}^{T}~\mathcal {Q}^{1}~\Psi ^{1}(t)\left( 1-\frac{{\mathscr {A}}^{T}~\mathcal {Q}^{1}~\Psi ^{1}(t)}{M_{1}}\right) +\gamma _{1}^{\eta _{1}}{\mathscr {D}}^{T}~\mathcal {Q}^{4}~\Psi ^{4}(t){\mathscr {A}}^{T}~\mathcal {Q}^{1}~\Psi ^{1}(t)\\ ~~~~~~~~~~-\frac{p_{1}^{\eta _{1}}{\mathscr {A}}^{T}~\mathcal {Q}^{1}~\Psi ^{1}(t){\mathscr {E}}^{T}~\mathcal {Q}^{5}~\Psi ^{5}(t)}{a_{1}+{\mathscr {A}}^{T}~\mathcal {Q}^{1}~\Psi ^{1}(t)},\\ {\mathscr {R}}_{2}(t)={\mathscr {B}}^{T}~\mathcal {Q}^{2}~\mathcal {D}^{\left( \eta _{2}\right) }_{t}~\Psi ^{2}(t)-k_{2}^{\eta _{2}}{\mathscr {A}}^{T}~\mathcal {Q}^{1}~\Psi ^{1}(t)\left( \frac{{\mathscr {A}}^{T}~\mathcal {Q}^{1}~\Psi ^{1}(t)}{M_{1}}\right) \left( 1-\frac{{\mathscr {B}}^{T}~\mathcal {Q}^{2}~\Psi ^{2}(t)}{M_{2}}\right) +n_{1}^{\eta _{2}}{\mathscr {B}}^{T}~\mathcal {Q}^{2}~\Psi ^{2}(t)\\ ~~~~~~~~~~+\gamma _{2}^{\eta _{2}}{\mathscr {D}}^{T}~\mathcal {Q}^{4}~\Psi ^{4}(t){\mathscr {A}}^{T}~\mathcal {Q}^{1}~\Psi ^{1}(t)-\frac{p_{2}^{\eta _{2}}{\mathscr {B}}^{T}~\mathcal {Q}^{2}~\Psi ^{2}(t){\mathscr {E}}^{T}~\mathcal {Q}^{5}~\Psi ^{5}(t)}{a_{2}+{\mathscr {B}}^{T}~\mathcal {Q}^{2}~\Psi ^{2}(t)},\\ {\mathscr {R}}_{3}(t)={\mathscr {C}}^{T}~\mathcal {Q}^{3}~\mathcal {D}^{\left( \eta _{3}\right) }_{t}~\Psi ^{3}(t)-q^{\eta _{3}}{\mathscr {C}}^{T}~\mathcal {Q}^{3}~\Psi ^{3}(t)\left( 1-\frac{{\mathscr {C}}^{T}~\mathcal {Q}^{3}~\Psi ^{3}(t)}{M_{3}}\right) +\delta ^{\eta _{3}} {\mathscr {C}}^{T}~\mathcal {Q}^{3}~\Psi ^{3}(t){\mathscr {B}}^{T}~\mathcal {Q}^{2}~\Psi ^{2}(t)\\ ~~~~~~~~~~+\frac{p_{3}^{\eta _{3}}{\mathscr {C}}^{T}~\mathcal {Q}^{3}~\Psi ^{3}(t){\mathscr {E}}^{T}~\mathcal {Q}^{5}~\Psi ^{5}(t)}{a_{3}+{\mathscr {C}}^{T}~\mathcal {Q}^{3}~\Psi ^{3}(t)},\\ {\mathscr {R}}_{4}(t)={\mathscr {D}}^{T}~\mathcal {Q}^{4}~\mathcal {D}^{\left( \eta _{4}\right) }_{t}~\Psi ^{4}(t)-s^{\eta _{4}}-\frac{\rho ^{\eta _{4}} {\mathscr {D}}^{T}~\mathcal {Q}^{4}~\Psi ^{4}(t){\mathscr {B}}^{T}~\mathcal {Q}^{2}~\Psi ^{2}(t)}{\omega +{\mathscr {B}}^{T}~\mathcal {Q}^{2}~\Psi ^{2}(t)}+\gamma _{3}^{\eta _{4}}{\mathscr {D}}^{T}~\mathcal {Q}^{4}~\Psi ^{4}(t){\mathscr {B}}^{T}~\mathcal {Q}^{2}~\Psi ^{2}(t)\\ ~~~~~~~~~~+n_{2}^{\eta _{4}}{\mathscr {D}}^{T}~\mathcal {Q}^{4}~\Psi ^{4}(t)+\frac{u^{\eta _{4}}{\mathscr {D}}^{T}~\mathcal {Q}^{4}~\Psi ^{4}(t){\mathscr {E}}^{T}~\mathcal {Q}^{5}~\Psi ^{5}(t)}{v+{\mathscr {E}}^{T}~\mathcal {Q}^{5}~\Psi ^{5}(t)},\\ {\mathscr {R}}_{5}(t)={\mathscr {E}}^{T}~\mathcal {Q}^{5}~\mathcal {D}^{\left( \eta _{5}\right) }_{t}~\Psi ^{5}(t)-\tau ^{\eta _{5}}+\left( \mu ^{\eta _{5}}+\frac{d_{1}^{\eta _{5}}{\mathscr {A}}^{T}~\mathcal {Q}^{1}~\Psi ^{1}(t)}{a_{1}+{\mathscr {A}}^{T}~\mathcal {Q}^{1}~\Psi ^{1}(t)}+\frac{d_{2}^{\eta _{5}}{\mathscr {B}}^{T}~\mathcal {Q}^{2}~\Psi ^{2}(t)}{a_{2}+{\mathscr {B}}^{T}~\mathcal {Q}^{2}~\Psi ^{2}(t)}+\frac{d_{3}^{\eta _{5}}{\mathscr {C}}^{T}~\mathcal {Q}^{3}~\Psi ^{3}(t)}{a_{3}+{\mathscr {C}}^{T}~\mathcal {Q}^{3}~\Psi ^{3}(t)}\right) {\mathscr {E}}^{T}~\mathcal {Q}^{5}~\Psi ^{5}(t). \end{array} \right. \end{aligned}$$

Meanwhile, from Eqs. (2.5) and (5.3), we have

5.5 $$\begin{aligned}&{\mathscr {A}}^{T}~\mathcal {Q}^{1}~\Psi ^{1}(0)-C(0)\triangleq \Theta _{1}\simeq 0,\quad {\mathscr {B}}^{T}~\mathcal {Q}^{2}~\Psi ^{2}(0)-T(0)\triangleq \Theta _{2}\simeq 0,\quad {\mathscr {C}}^{T}~\mathcal {Q}^{3}~\Psi ^{3}(0)-H(0)\triangleq \Theta _{3}\simeq 0,\nonumber \\&{\mathscr {D}}^{T}~\mathcal {Q}^{4}~\Psi ^{4}(0)-I(0)\triangleq \Theta _{4}\simeq 0,\quad {\mathscr {E}}^{T}~\mathcal {Q}^{5}~\Psi ^{5}(0)-E(0)\triangleq \Theta _{5}\simeq 0. \end{aligned}$$

We can generate the 2-norm of the residual functions as

5.6 $$\begin{aligned} \mathcal {M}(\mathcal {Q}^{1}, \mathcal {Q}^{2}, \mathcal {Q}^{3}, \mathcal {Q}^{4}, \mathcal {Q}^{5}, \Phi ^{i})=\int _{0}^{k}\bigg ({\mathscr {R}}_{1}^2+{\mathscr {R}}_{2}^2+{\mathscr {R}}_{3}^2+{\mathscr {R}}_{4}^2+{\mathscr {R}}_{5}^2\bigg )(t)dt, \end{aligned}$$

where $\mathcal {Q}^{i}$ and $\Phi ^{i}$, $i=1,2,\ldots ,5$, are the free coefficients and control parameters, respectively.

To obtain the optimal values for the free coefficients and control parameters, we consider the following optimization problem

5.7 $$\begin{aligned} \min \,\mathcal {M}(\mathcal {Q}^{1}, \mathcal {Q}^{2}, \mathcal {Q}^{3}, \mathcal {Q}^{4}, \mathcal {Q}^{5}, \Phi ^{i}), \end{aligned}$$

subject to Eq. (5.5), where $\mathcal {M}$ is the objective function.

To solve this minimization problem, we assume that

5.8 $$\begin{aligned} \mathcal {J}[\mathcal {Q}^{1}, \mathcal {Q}^{2}, \mathcal {Q}^{3}, \mathcal {Q}^{4}, \mathcal {Q}^{5}, \Phi ^{i},\lambda ]= \mathcal {M}(\mathcal {B}^{1}, \mathcal {B}^{2}, \mathcal {B}^{3}, \mathcal {B}^{4}, \mathcal {B}^{5}, \Phi ^{i})+\lambda \Theta , \end{aligned}$$

where $\lambda$ denotes unknown Lagrange multipliers.

In order to obtain the extremum, the necessary conditions are:

5.9 $$\begin{aligned} \displaystyle \left\{ \begin{array}{lll} \displaystyle \displaystyle \frac{\partial \mathcal {J}}{\partial \lambda }=0,\\ \displaystyle \frac{\partial \mathcal {J}}{\partial \mathcal {Q}^{i}}=0,\frac{\partial \mathcal {J}}{\partial \Phi ^{i}}=0,&{}&{}i=0,1,\ldots ,5. \end{array}\right. \end{aligned}$$

Finally, by solving Eq. (5.9) using a software package to calculate the components $\mathcal {Q}^{1}$, $\mathcal {Q}^{2}$, $\mathcal {Q}^{3}$, $\mathcal {Q}^{4}$, $\mathcal {Q}^{5}$, $\Phi ^{i}$ and $\lambda$, we obtain the approximate solutions *C*(*t*), *T*(*t*), *H*(*t*), *I*(*t*) and *E*(*t*) of Eq. (3.5). In this study we used Maple 18 (with 20 digits precision) for the above extracted system and also for all numerical simulations. The step-by-step algorithm of the new technique is summarised as follows: 
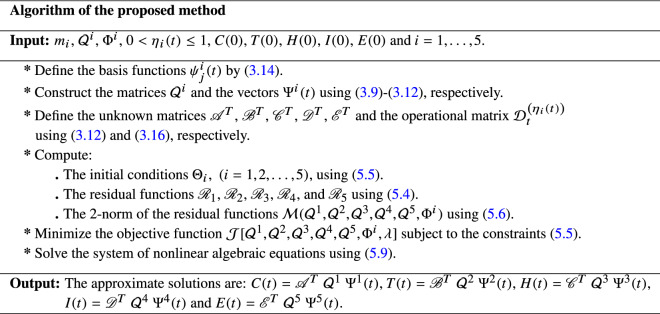


## Numerical simulations

In this section, two examples are considered to illustrate the applicability of the proposed technique when solving the F-BCCM (2.5). The numerical simulations are based on the packages Maple 18 (with accuracy 40 decimal digits) and Matlab.

### Example 1

**Figure 1:**
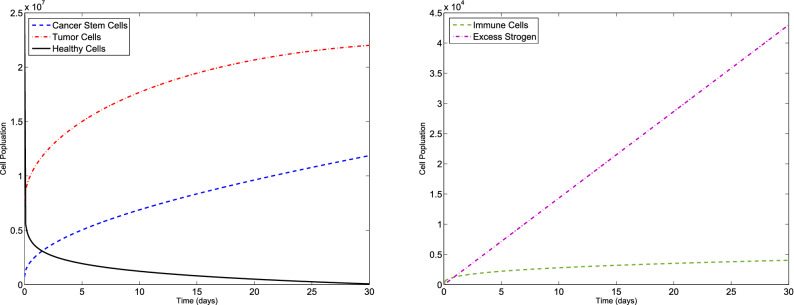
Evolution of $\{C(t), T(t), H(t), I(t), E(t)\}$ vs time for the F-BCCM with $m_{1}=2$, $m_{2}=m_{3}=m_{4}=3$, $m_{5}=4$, $\eta _{1}=0.10$, $\eta _{2}=0.15$, $\eta _{3}=0.20$, $\eta _{4}=0.25$ and $\eta _{5}=0.30$ for Example 1.

**Figure 2:**
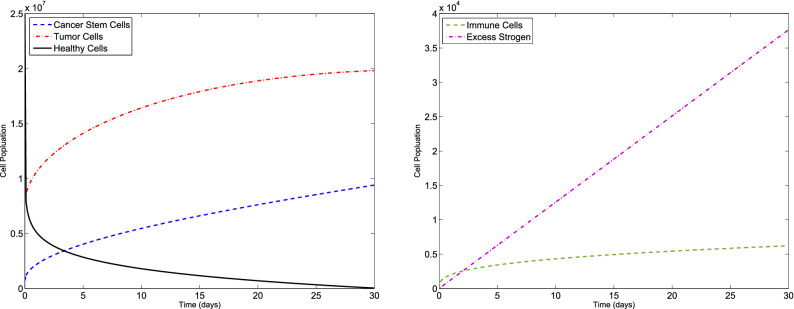
Evolution of $\{C(t), T(t), H(t), I(t), E(t)\}$ vs time for the F-BCCM with $m_{1}=2$, $m_{2}=m_{3}=m_{4}=3$, $m_{5}=4$, $\eta _{1}=0.98$, $\eta _{2}=0.99$, $\eta _{3}=0.97$, $\eta _{4}=0.98$ and $\eta _{5}=0.99$ for Example 1.

**Figure 3:**
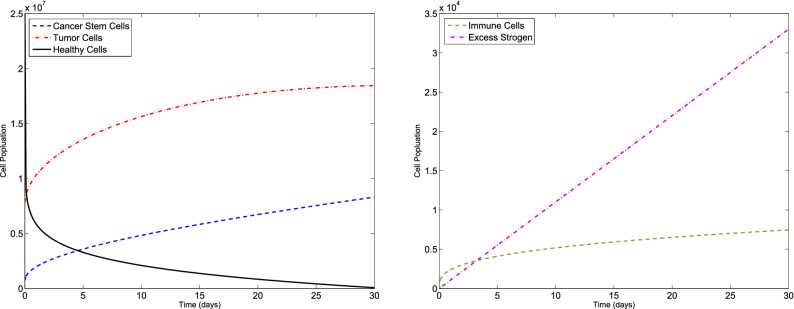
Evolution of $\{C(t), T(t), H(t), I(t), E(t)\}$ vs time for the F-BCCM with $m_{1}=3$, $m_{2}=m_{3}=m_{4}=4$ and $m_{5}=5$, $\eta _{1}=0.10$, $\eta _{2}=0.15$, $\eta _{3}=0.20$, $\eta _{4}=0.25$ and $\eta _{5}=0.30$ for Example 1.

**Figure 4:**
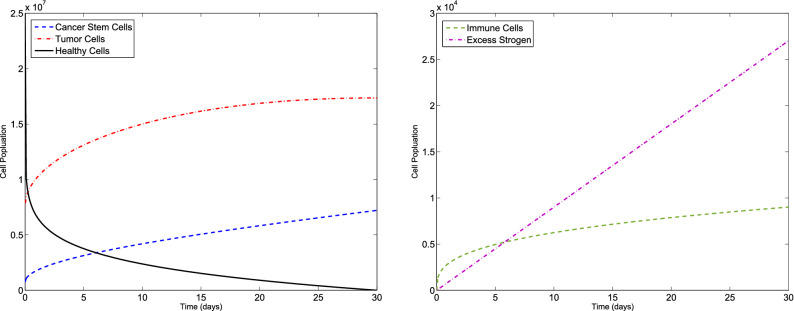
Evolution of $\{C(t), T(t), H(t), I(t), E(t)\}$ vs time for the F-BCCM with $m_{1}=3$, $m_{2}=m_{3}=m_{4}=4$ and $m_{5}=5$, $\eta _{1}=0.98$, $\eta _{2}=0.99$, $\eta _{3}=0.97$, $\eta _{4}=0.98$ and $\eta _{5}=0.99$ for Example 1.

Consider the F-BCCM (2.5) and the parameters listed in Table 1. The algorithm is used to solve the F-BCCM (2.5) with $C(0)=7.3710 \times 10^{5}$, $T(0)=7.6167\times 10^{6}$, $H(0)=2.5000\times 10^{7}$, $I(0)=0$ and $E(0)=0$, for different values of $\eta _{i}$ and $m_{i}$, $i=1,2,3,4,5$. The approximate solutions of the five state variables $\{C(t), T(t), H(t), I(t), E(t)\}$, with $m_{1}=2$, $m_{2}=m_{3}=m_{4}=3$, $m_{5}=4$, for $\eta _{1}=0.10$, $\eta _{2}=0.15$, $\eta _{3}=0.20$, $\eta _{4}=0.25$, $\eta _{5}=0.30$ and also $\eta _{1}=0.98$, $\eta _{2}=0.99$, $\eta _{3}=0.97$, $\eta _{4}=0.98$, $\eta _{5}=0.99$ are shown in Figs. 1 and 2, respectively. The plots of the approximate solutions with $m_{1}=3$, $m_{2}=m_{3}=m_{4}=4$, $m_{5}=5$ for $\eta _{1}=0.10$, $\eta _{2}=0.15$, $\eta _{3}=0.20$, $\eta _{4}=0.25$, $\eta _{5}=0.30$ and also $\eta _{1}=0.98$, $\eta _{2}=0.99$, $\eta _{3}=0.97$, $\eta _{4}=0.98$, $\eta _{5}=0.99$ are illustrated in Figs. 3 and 4, respectively. The runtime of the proposed method and the optimal values of the residual function with different choices of $m_{i}$, $i=1,2,3,4,5$ are reported in Tables 2 and 3, respectively. According to Figs. 1, 2, 3, 4, the number of cancer stem and tumor cells and estrogen, *C*, *T* and *E*, decreases, while the healthy and immune cells, *H* and *I*, increase. This effect is associated with better prognosis and longer patient survival. The obtained results with the proposed method provide a meaningful solution by applying even a small number of basis functions. From Table 2 we verify that if we select large values of $m_{i}$, $i=1,2,3,4,5$, then we pose an higher computational load.

**Table 2:** The runtime (in seconds) of the proposed method with different choices of $m_{i}$, $i=1,2,3,4,5$, for Example 1.

Case	$m_{1}$	$m_{2}$	$m_{3}$	$m_{4}$	$m_{5}$	CPU time	CPU time
						$\eta _{1}=0.10,\eta _{2}=0.15,\eta _{3}=0.20,\eta _{4}=0.25,\eta _{5}=0.30$	$\eta _{1}=0.98,\eta _{2}=0.99,\eta _{3}=0.97,\eta _{4}=0.98,\eta _{5}=0.99$
1	2	3	3	3	4	15.23	15.29
2	3	4	4	4	5	19.74	19.76

**Table 3:** The optimal values of the residual function with different values of $m_{i}$, $i=1,2,3,4,5$, for Example 1.

Case	$m_{1}$	$m_{2}$	$m_{3}$	$m_{4}$	$m_{5}$	Residual function	Residual function
						$\eta _{1}=0.10,\eta _{2}=0.15,\eta _{3}=0.20,\eta _{4}=0.25,\eta _{5}=0.30$	$\eta _{1}=0.98,\eta _{2}=0.99,\eta _{3}=0.97,\eta _{4}=0.98,\eta _{5}=0.99$
1	2	3	3	3	4	$7.4167E-07$	$2.9968E-07$
2	3	4	4	4	5	$8.2858E-08$	$7.4587E-09$

### Example 2

**Figure 5:**
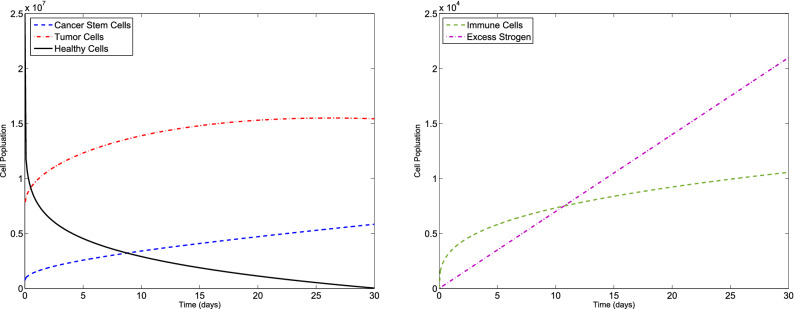
Evolution of $\{C(t), T(t), H(t), I(t), E(t)\}$ vs time for the F-BCCM with $m_{1}=m_{2}=4$, $m_{3}=5$, $m_{4}=m_{5}=7$, $\eta _{1}=0.08$, $\eta _{2}=0.17$, $\eta _{3}=0.13$, $\eta _{4}=0.11$ and $\eta _{5}=0.23$ for Example 2.

**Figure 6:**
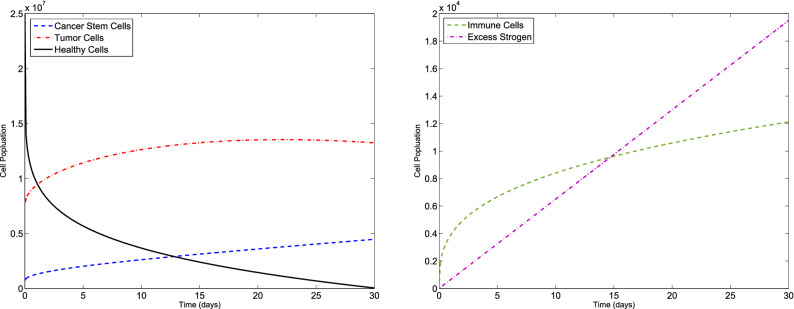
Evolution of $\{C(t), T(t), H(t), I(t), E(t)\}$ vs time for the F-BCCM with $m_{1}=m_{2}=4$, $m_{3}=5$, $m_{4}=m_{5}=7$, $\eta _{1}=0.96$, $\eta _{2}=0.98$, $\eta _{3}=0.99$, $\eta _{4}=0.96$ and $\eta _{5}=0.95$ for Example 2.

**Figure 7:**
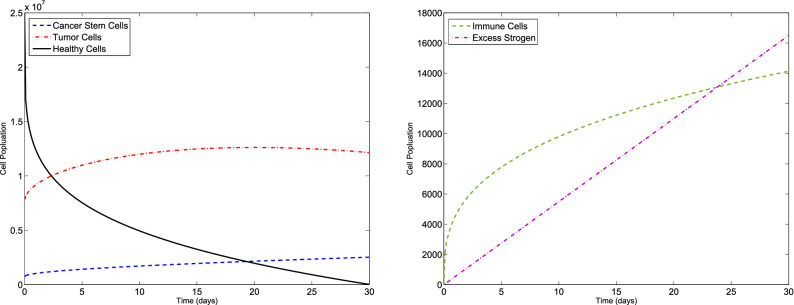
Evolution of $\{C(t), T(t), H(t), I(t), E(t)\}$ vs time for the F-BCCM with $m_{1}=m_{2}=m_{3}=6$, $m_{4}=m_{5}=8$, $\eta _{1}=0.08$, $\eta _{2}=0.17$, $\eta _{3}=0.13$, $\eta _{4}=0.11$ and $\eta _{5}=0.23$ for Example 2.

**Figure 8:**
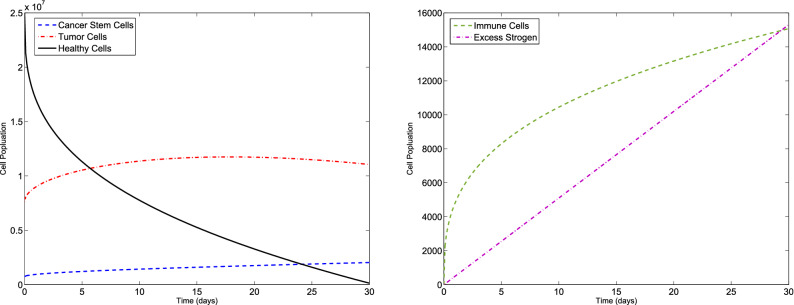
Evolution of $\{C(t), T(t), H(t), I(t), E(t)\}$ vs time for the F-BCCM with $m_{1}=m_{2}=m_{3}=6$, $m_{4}=m_{5}=8$, $\eta _{1}=0.96$, $\eta _{2}=0.98$, $\eta _{3}=0.99$, $\eta _{4}=0.96$ and $\eta _{5}=0.95$ for Example 2.

Let us consider the F-BCCM (2.5) and the parameters values given in Table 1. We consider $C(0)=7.3710 \times 10^{5}$, $T(0)=7.6167\times 10^{6}$, $H(0)=2.5000\times 10^{7}$, $I(0)=0$ and $E(0)=0$ for different values of $\eta _{i}$ and $m_{i}$, $i=1,2,3,4,5$. The new method is applied to obtain the numerical solution of the F-BCCM (2.5). The approximate solutions of the five state variables $\{C(t), T(t), H(t), I(t), E(t)\}$ with $m_{1}=m_{2}=4$, $m_{3}=5$ and $m_{4}=m_{5}=7$ for $\eta _{1}=0.08$, $\eta _{2}=0.17$, $\eta _{3}=0.13$, $\eta _{4}=0.11$, $\eta _{5}=0.23$ and also $\eta _{1}=0.96$, $\eta _{2}=0.98$, $\eta _{3}=0.99$, $\eta _{4}=0.96$, $\eta _{5}=0.95$ are shown in Figs. 5 and 6, respectively. The plots of the approximate solutions with $m_{1}=m_{2}=m_{3}=6$, $m_{4}=m_{5}=8$ for $\eta _{1}=0.08$, $\eta _{2}=0.17$, $\eta _{3}=0.13$, $\eta _{4}=0.11$, $\eta _{5}=0.23$ and also $\eta _{1}=0.96$, $\eta _{2}=0.98$, $\eta _{3}=0.99$, $\eta _{4}=0.96$, $\eta _{5}=0.95$ are illustrated in Figs. 7 and 8, respectively. The runtime of the proposed method and the optimal values of the residual function with different choices of $m_{i}$, $i=1,2,3,4,5$, are reported in Tables 4 and 5, respectively. According to Figs. 5, 6, 7, 8, the number of cancer stem and tumor cells and estrogen, *C*, *T* and *E*, decreases, while the healthy and immune cells, *H* and *I*, increase. This effect is associated with better prognosis and longer patient survival. Also, from Table 4 we conclude that if we choose large values of $m_{i}$, $i=1,2,3,4,5$, then leads to an higher computational load.

**Table 4:** The runtime (in seconds) of the proposed method with different choices of $m_{i}$, $i=1,2,3,4,5$, for Example 2.

Case	$m_{1}$	$m_{2}$	$m_{3}$	$m_{4}$	$m_{5}$	CPU time	CPU time
						$\eta _{1}=0.08,\eta _{2}=0.17,\eta _{3}=0.13,\eta _{4}=0.11,\eta _{5}=0.23$	$\eta _{1}=0.96,\eta _{2}=0.98,\eta _{3}=0.99,\eta _{4}=0.96,\eta _{5}=0.95$
1	4	4	5	7	7	26.41	26.43
2	6	6	6	8	8	35.08	35.18

**Table 5:** The optimal values of the residual function with different choices of $m_{i}$, $i=1,2,3,4,5$, for Example 2.

Case	$m_{1}$	$m_{2}$	$m_{3}$	$m_{4}$	$m_{5}$	Residual function	Residual function
						$\eta _{1}=0.08,\eta _{2}=0.17,\eta _{3}=0.13,\eta _{4}=0.11,\eta _{5}=0.23$	$\eta _{1}=0.96,\eta _{2}=0.98,\eta _{3}=0.99,\eta _{4}=0.96,\eta _{5}=0.95$
1	4	4	5	7	7	$3.9316E-11$	$4.8842E-12$
2	6	6	6	8	8	$8.0023E-14$	$1.1164E-14$

### Remark 2

Abernathy et al.^19^ presented a system of five ordinary differential equations which consider the population dynamics among cancer stem, tumor, and healthy cells. They described (i) the effects of excess estrogen and the body’s natural immune response on the aforementioned cell populations and (ii) the global dynamics of the F-BCCM (2.5), with $\eta _{i}=1$, $i=1,2,3,4,5$, along with various submodels by employing a variety of analytical methods. In this paper we consider the integer order model (2.1) proposed initially in^19^ and generalize it to fractional order (2.5). Additionally, we introduced a new basis function (i.e., the GSLP) for solving the F-BCCM (2.5) and obtained optimally the unknoun coefficients and parameters. From the results we verify not only that the approximation by the new method provides good approximate solutions, but also that it is superior to the one proposed by Abernathy et al.^19^.

## Epidemiologic and clinical relevance

Tumor cells proliferate abnormally and gradually undergo changes that induce the growth and development of cancer with an high mutation rate and spread, leading to tumor progression. A small number of cancer stem cells can be the source of cancer and cause recurrence, metastasis and resistance to treatment. Presently, stem cells are being targeted for cancer treatment, so that with a lower number of cancer cell we can expect better prognosis. On the other hand, healthy cells must exhibit a normal proliferation and function. In other words, they do not undergo aberrant proliferation and malignant changes. Immune cells play a key role in defending the body against foreign agents and deformed cells such as those of cancer. An high number of immune cells, in particular of *T* lymphocytes, correlates with a better prognosis for the patient. Epidemiological studies revealed the pivotal role of estrogen in the initiation and progression of breast cancer^60^. The hormone has also influence in the mechanism of some drugs for treatment, since they inhibit estrogen and the duration of exposure to the estrogen increases the risk of breast cancer^61–63^. During the progression of the breast cancer there are evidences that an increasing number of immune cell infiltrate in tumor parenchyma, including the cytotoxic CD8$^+$ T, CD4$^+$ T helper, B, macrophages and dendritic cells, the natural killer cells, and cytokines, such as interferons, interleukins, chemokines and growth factors^64,65^. Immune cells contain estrogen receptors and are regulated by estrogens as well. Therefore, strogens could influence immune cells in breast cancer^64^. Parenchymal and stromal cells of breast may be accessible to several immune cells subtypes that lead to decreasing the tumor cells and reducing tumor growth^64^. Therefore, from the medical point of view, the numerical results show that the approximate solution is coherent with real-word experience.

From the numerical viewpoint, we must highlight that there is a basic difference between the proposed approach and other spectral methods. In fact, the main idea of spectral methods (based on the Legendre, Chebyshev, Lagrange and Jacobi polynomials) is to express the solution of a differential equation as a sum of the basis functions and then to choose the coefficients in order to minimize the error between the numerical and exact solutions, in some suitable sense. To determine the coefficients, three main techniques, are commonly employed, namely the Galerkin, tau and collocation methods. In the present case, the residual function and its 2-norm are employed for converting the problem to an optimization one, so that the unknown parameters are obtained optimally. As a result, the necessary conditions of optimality are derived in the form of a system of nonlinear algebraic equations with unknown parameters. It is also worth mentioning that approximating any arbitrary smooth function by the eigenfunctions of singular Sturm-Liouville problems, such as the Legendre, Chebyshev, Hermite, Lagrange, Laguerre or Jacobi polynomials, has $''$spectral accuracy$''$. This means that the truncation error approaches zero faster than any negative power of the number of the basis functions used in the approximation, as that number tends to infinity. Consequently, these basis functions are not the most adequate for approximating non analytic functions, in contrast which occurs when using the GSLP which prove to be much more efficient.

## Conclusion

This paper developed and analyzed the GSLP method for solving the F-BCCM. The proposed approach is based on the operational matrices of the GSLP and the Lagrange multipliers. By adopting the GSLP basis and operational matrices of F-CD, the problem was reduced to the solution of a system of algebraic equations. The convergence analysis for the new algorithm was also carried out. Two numerical examples illustrate the ability and reliability of the algorithm. This method shows that with fewer number of basis functions we can obtain the approximate the solutions. This model and algorithm can be further explored to develop in silico studies of the dynamics and cancer problems.
